# Relative Contributions of Agricultural Drift, Para-Occupational, and Residential Use Exposure Pathways to House Dust Pesticide Concentrations: Meta-Regression of Published Data

**DOI:** 10.1289/EHP426

**Published:** 2016-07-26

**Authors:** Nicole C. Deziel, Laura E. Beane Freeman, Barry I. Graubard, Rena R. Jones, Jane A. Hoppin, Kent Thomas, Cynthia J. Hines, Aaron Blair, Dale P. Sandler, Honglei Chen, Jay H. Lubin, Gabriella Andreotti, Michael C. R. Alavanja, Melissa C. Friesen

**Affiliations:** 1 Division of Cancer Epidemiology and Genetics, National Cancer Institute, National Institutes of Health, Department of Human Health and Services, Bethesda, Maryland, USA; 2 Department of Environmental Health Sciences, Yale School of Public Health, Yale University, New Haven, Connecticut, USA; 3 Department of Biological Sciences, Center for Human Health and the Environment, North Carolina State University, Raleigh, North Carolina, USA; 4 National Exposure Research Laboratory, U.S. Environmental Protection Agency, Research Triangle Park, North Carolina, USA; 5 Division of Surveillance, Hazard Evaluations and Field Studies, National Institute for Occupational Safety and Health, Cincinnati, Ohio, USA; 6 Epidemiology Branch, National Institute of Environmental Health Sciences, National Institutes of Health, Department of Health and Human Services, Research Triangle Park, North Carolina, USA

## Abstract

**Background:**

Increased pesticide concentrations in house dust in agricultural areas have been attributed to several exposure pathways, including agricultural drift, para-occupational, and residential use.

**Objective:**

To guide future exposure assessment efforts, we quantified relative contributions of these pathways using meta-regression models of published data on dust pesticide concentrations.

**Methods:**

From studies in North American agricultural areas published from 1995 to 2015, we abstracted dust pesticide concentrations reported as summary statistics [e.g., geometric means (GM)]. We analyzed these data using mixed-effects meta-regression models that weighted each summary statistic by its inverse variance. Dependent variables were either the log-transformed GM (drift) or the log-transformed ratio of GMs from two groups (para-occupational, residential use).

**Results:**

For the drift pathway, predicted GMs decreased sharply and nonlinearly, with GMs 64% lower in homes 250 m versus 23 m from fields (interquartile range of published data) based on 52 statistics from seven studies. For the para-occupational pathway, GMs were 2.3 times higher [95% confidence interval (CI): 1.5, 3.3; 15 statistics, five studies] in homes of farmers who applied pesticides more recently or frequently versus less recently or frequently. For the residential use pathway, GMs were 1.3 (95% CI: 1.1, 1.4) and 1.5 (95% CI: 1.2, 1.9) times higher in treated versus untreated homes, when the probability that a pesticide was used for the pest treatment was 1–19% and ≥ 20%, respectively (88 statistics, five studies).

**Conclusion:**

Our quantification of the relative contributions of pesticide exposure pathways in agricultural populations could improve exposure assessments in epidemiologic studies. The meta-regression models can be updated when additional data become available.

**Citation:**

Deziel NC, Beane Freeman LE, Graubard BI, Jones RR, Hoppin JA, Thomas K, Hines CJ, Blair A, Sandler DP, Chen H, Lubin JH, Andreotti G, Alavanja MC, Friesen MC. 2017. Relative contributions of agricultural drift, para-occupational, and residential use exposure pathways to house dust pesticide concentrations: meta-regression of published data. Environ Health Perspect 125:296–305; http://dx.doi.org/10.1289/EHP426

## Introduction

Adults living in agricultural areas may be exposed to pesticides from several occupational and environmental sources and pathways (Arbuckle et al. 2006; Curl et al. 2002; Deziel et al. 2015b; Harnly et al. 2009; Ward et al. 2006). Understanding the contribution of these use and transport exposure pathways to overall exposure is necessary for developing exposure assessment approaches for epidemiologic studies, designing exposure studies in nonoccupationally exposed populations, and for developing effective risk mitigation strategies. To provide a surrogate for quantitative, long-term, multi-source indoor pesticide exposure, pesticide concentrations in house dust have been measured in many studies (Butte and Heinzow 2002; Deziel et al. 2013; Lioy et al. 2002; Colt et al. 2004), in part because many pesticide biomarkers have short half-lives (measured in hours) reflecting recent exposure (Barr et al. 2006). Our recent qualitative review of exposure studies in North American agricultural environments found that increased pesticide concentrations in house dust were associated with take-home exposure from closer distances between homes and treated fields (agricultural drift pathway), farm work by one or more house residents (para-occupational pathway), and greater residential use of pesticides to treat various home, garden, and yard insects and weeds (residential use pathway) (Deziel et al. 2015b). To quantify the magnitude of these effects, we analyzed the house dust pesticide concentrations reported in published studies to obtain a summary measure of effect for each pathway across multiple pesticides and studies.

The analysis of the published data, however, presented a statistical challenge because the published dust pesticide concentrations were reported as summary statistics (i.e., means) or ratios (i.e., predicted relative difference obtained from regression models) rather than individual measurements. As a result, we needed to account for both the number of measurements and their variability to obtain an accurate summary effect measure. Koh et al. (2014) recently demonstrated the utility of mixed-effects meta-regression models to handle this challenge in an analysis synthesizing published lead exposure data to obtain temporal trends in occupational lead exposure. Our primary aim was to quantify the relative magnitude of exposure differences in dust pesticide concentrations in relation to surrogates representing each of the agricultural drift (e.g., distance of house to fields), para-occupational (e.g., how frequently a household member applies pesticides agriculturally), and residential pesticide use (e.g., treatment of insects or weeds in the home, yard, or garden) exposure pathways in North American agricultural homes. We focused on relative, rather than absolute, differences in dust pesticide concentrations within a pathway so that we could model the relationships across multiple active ingredients, for which absolute concentrations varied by orders of magnitude. Our secondary aim, undertaken to address the statistical challenges encountered, was to extend a mixed-effects meta-regression modeling approach used previously for epidemiologic analyses and occupational exposure data to environmental exposure data. To our knowledge, this represents one of the first uses of meta-regression models to synthesize published environmental measurements across multiple studies (Bain et al. 2014; Shields et al. 2015).

## Methods

### Data Abstraction

We included publications reporting pesticide concentrations in house dust in relation to agricultural drift, para-occupational activities, or residential use of pesticides in North American agricultural homes from our prior literature review (Deziel et al. 2015b) and one study that was published subsequent to our review (Deziel et al. 2015a). The prior systematic search identified studies published through September 2013 mainly from a PubMed search with the following terms: “environmental exposure [MeSH] AND pesticides [MeSH] AND (home OR household OR indoor).” We also searched Scopus, Web of Science, and Google Scholar, and examined reference lists of relevant publications. For the current analysis, we selected studies that measured pesticide concentrations in house dust because dust measurements are used as proxies for long-term environmental exposure (Butte and Heinzow 2002; Deziel et al. 2013). Based on findings from our prior review, we excluded studies with only air, food, or water samples due to low pesticide detection rates for those measures, and we excluded biological measurements because the measured pesticide biomarkers tended to have low percent detection and limited variability, and generally reflected only recent exposure. We repeated the PubMed search in March 2015 and identified one additional publication meeting the above criteria. Overall, 10 studies with published house dust pesticide concentrations were included.

From each study related to the agricultural drift pathway, we abstracted summary statistics of the house dust pesticide concentrations and the distances between the homes and the nearest fields. For distances reported categorically, we assigned the midpoint of the category. We used units of feet, because it was the most commonly reported unit and coincides with the response categories in many U.S. studies, including the Agricultural Health Study.

From each study related to the para-occupational pathway, we abstracted summary statistics of the dust pesticide concentrations for independent groups with different exposure potential (“comparison groups”). We extracted data for farmers with high pesticide use (high use group) versus low pesticide use (reference group), based on the frequency and recentness of pesticide application. In three studies, the high use group was farmers who applied pesticides generally and the reference group was those who did not apply pesticides (Fenske et al. 2002; Lu et al. 2000; Simcox et al. 1995). For two studies, both the high use and reference groups included pesticide applicators; therefore the comparison groups were those who applied the pesticide of interest either within 7 or 30 days of sampling (recentness of application varied by the pesticide active ingredient) versus those who did not apply the pesticide of interest within 7 or 30 days of sampling (Curwin et al. 2005), or those who applied atrazine ≥ 2 days/season versus < 2 days/season (Golla et al. 2012).

From studies of the residential use pathway, we extracted data from agricultural households reporting specific pest treatments (high use groups) and households reporting no treatment for that pest (reference group). In these studies, homeowners reported the type of pest treatment but did not provide the active ingredients of those treatments. Therefore, we extracted the type of pest treatment (e.g., fleas/ticks, weeds) and then derived a probability assessment of whether the treatment type was associated with the measured active ingredient using the National Cancer Institute (NCI) Pesticide Exposure Matrix (http://dceg.cancer.gov/tools/design/pesticide) (Colt et al. 2007). This publicly available tool uses product sales and label information to predict the probability that an active ingredient was used in 96 different scenarios [12 pest treatment types, whether the applicator was a general consumer or professional commercial applicator, and four time frames (1976, 1980, 1990, 2000)]. We assigned the probabilities from the time frame closest to that of the individual study and, if multiple scenarios were relevant, averaged their probabilities. For example, from studies of weed treatment of lawns, we averaged the probabilities from the “professional weeds” and “consumer weeds” scenarios. We categorized the probabilities as 0% (active ingredient not listed), 1–19%, and ≥ 20%.

From the 10 studies for the above-mentioned comparison groups, we abstracted the available information on dust pesticide concentrations. These data were predominantly reported as summary results [i.e., arithmetic means (AMs), geometric means (GMs), standard deviations (SDs), geometric standard deviations (GSDs), number of measurements (N)]. We also abstracted data on the ratios between two comparison groups predicted from multivariable regression models, rather than GMs. These data were usually reported in tables; however, we also extracted data from boxplots and other figures when necessary. For each set of summary statistics, we obtained reported ancillary data, including study years, pesticide active ingredient, pesticide type (e.g., herbicide, insecticide, fungicide), and crop type (e.g., corn, orchard fruit). If the same measurements were reported both in descriptive analyses and in multivariable regression models within the same paper, we abstracted the data only once. To best capture the independent contribution of a single pathway, we abstracted data that accounted for the other potential pathways through adjustment in multivariable regression models or stratification wherever possible.

### Data Treatment

We obtained a GM and GSD for each set of published dust pesticide concentrations. When these summary statistics were not directly reported, we estimated them using available formulae presented in Equations 1 through 6, where *AM* is the arithmetic mean, *SD* is the standard deviation, *max* is the maximum value, *min* is the minimum value, *p25* is the value at the 25th percentile, and *p10* is the value at the 10th percentile (Hein et al. 2008; Hewett 2005; Koh et al. 2014; Lavoué et al. 2007; Aitchison and Brown 1963).


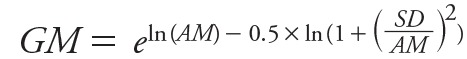
 [1]

*GM* = *median* [2]

*GM* = *e* ^(ln(^ *^max^* ^) + ln(^ *^min^* ^))/2^ [3]


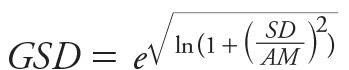
 [4]

*GSD* = (*e* ^(ln(^ *^p25^* ^) – ln(^ *^GM^* ^))/–0.68^ + *e* ^(ln(^ *^p10^* ^) – ln(^ *^GM^* ^))/–1.282^)/2 [5]

*GSD* = *e* ^(ln(^ *^max^* ^) – ln(^ *^min^* ^))/4^ [6]

Two studies collected more than one sample per home (Curwin et al. 2005; Golla et al. 2012). We accounted for the repeated within-home measurements in these studies by adjusting the number of samples collected using a design effect (Equation 7) to calculate the effective sample size (Equation 8) (Kish 1965). We divided the between-home variance by the sum of the between- and within-home variances to obtain the intra-class correlation coefficient (ICC) for each active ingredient (Curwin et al. 2005). In these two studies, the calculated effective sample size replaced the total number of measurements (*N_samples_*) for each summary statistic, where *N_homes_* is the number of homes corresponding to each summary statistic.


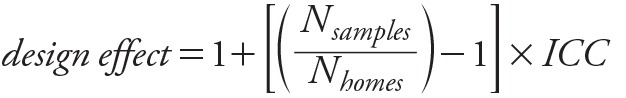
 [7]

*effective sample size* = *N_samples_*/*design effect* [8]

The GMs of the data on the effect of agricultural drift were approximately log-normally distributed based on visual inspection and therefore were natural log-transformed prior to additional analyses. For these data, we calculated the variance of each log-transformed GM using Equation 9, which we derived for these analyses using the delta method:


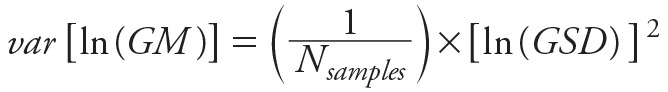
 [9]

For the para-occupational and residential use pathways, the data were often abstracted from multivariable regression models that examined the association between log-transformed exposure and various determinants of exposure. We interpreted the anti-log of a model parameter, exp(β), as the ratio between the GMs of the high use group and the reference group. Hence, to include these data, we assumed that β equaled ln(ratio) and the β’s standard error squared (SE^2^) equaled the variance of the ln(ratio). When the standard error was not reported, we extracted it from the lower and upper confidence limits (*LCL* and *UCL*) of the exp(β) using Equation 10. Note that Equation 10 assumes that the LCL and UCL were reported as exponentiated terms, as was the case in these studies, and thus required transformation back to the log-scale.


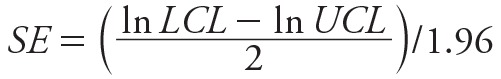
 [10]

To combine these regression parameter statistics with the data that were abstracted as GMs required converting the GMs to ratios. For these two pathways, we calculated the ratio of the GMs of the high use group compared to the reference group (Equation 11). The ratios were assumed log-normally distributed based on visual inspection and log-transformed prior to additional analyses. For these data we calculated the variance of the ratio of the log-transformed GMs using Equation 12, which we derived using the delta method.


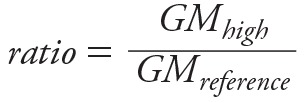
 [11]


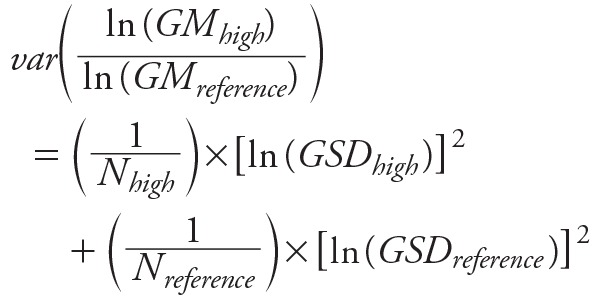
 [12]

### Meta-Regression Models

We developed separate mixed-effects meta-regression models for each of the agricultural drift, para-occupational, and residential use pathways using PROC MIXED (version 9.3; SAS Institute Inc., Cary, NC). In these models, each summary statistic was weighted by the inverse of its study-specific variance. Regression parameters and between-study variances were obtained using maximum likelihood estimation. Estimation of the between-study variance required a starting value for computation, which we set to one-half of the mean within-study variance (Konstantopoulos and Hedges 2004). Pathway-specific analyses are described below, with the SAS code for each pathway’s primary model provided in the Supplemental Material, Appendix 1. For each pathway, we examined how the relative magnitude of dust pesticide concentrations varied based on surrogate measures, such as the relative change in dust pesticide concentrations at varying distances of the house to fields for the agricultural drift pathway. Exposure comparisons were made within, but not across pathways.

For agricultural drift, the dependent variable was the log-transformed GM. Most studies included GMs at various distances from treated fields and the reference distance varied between studies. As a result, the agricultural drift model incorporated two random effects: one identified each unique combination of publication, active ingredient, and distance from field to weight each statistic by the inverse of its study-specific variance and a second identified each unique combination of publication and active ingredient to account for active ingredient- and study-specific differences in baseline pesticide concentrations. We identified the best parametric characterization between the log-transformed GM and distance by evaluating various forms, including linear distance, natural log-transformed distance, inverse distance, and inverse distance squared. The natural log-transformed distance provided the best model fit based on the Akaike information criterion that was also consistent with graphical evaluations (not shown). In preliminary models, we tested a random slope for the relationship between ln(GM) and ln(distance) to allow for study- and pesticide-specific differences in the slope; and found no differences in slopes, although data were sparse. The overall drift model included only ln(distance) and provided an estimate of the GM dust pesticide concentration at varying distances in ft (*d*) of the house from the fields using the regression coefficients for the intercept (β*_intercept_*) and ln(distance) (β*_slope_*) (Equation 13). The percent change in GMs between two specific distances (*d*_1_ and *d*_2_) is calculated using Equation 14. In sensitivity analyses, we also developed separate models for herbicides, insecticides, and chlorpyrifos (the most commonly measured insecticide).

*Predicted GM* = exp[β*_intercept_ +* β*_slope_ × ln*(*d*)] = *d* ^β^ *^_slope_^ e* ^(β^ *^_intercept_^* ^)^ [13]

*% change in GM between d*_1_ and *d*_2_  = [(*GM_d_*__1__ – *GM_d_*__2__)/*GM_d_*__1__] × 100  = [1 – (*d*_2_/*d*_1_)^β^ *^_slope_^*] *×* 100 [14]

For para-occupational and residential use, the data were often abstracted as ratios from multivariable regression models, and these models used the log-transformed ratios of GMs from the high use and reference groups as the dependent variable. Using the ratio had the added benefit of removing the active-ingredient-specific differences in baseline; thus these models incorporated a single random intercept, which identified each unique combination of publication and pesticide active ingredient to weight each observation by the inverse of its study-specific variance.

For para-occupational exposure, we calculated an overall summary of the effect of the take-home pathway. In sensitivity analyses, we also developed separate models for the two types of comparison groups (farmers who ever applied vs. never applied pesticides and farmers who applied the pesticides in specific time windows or with specific frequencies). Studies comparing farmers who ever applied versus never applied pesticides were mainly of insecticide applications to fruit orchards; whereas, the studies comparing groups with more specific timing or frequency of applications addressed herbicide applications to row crops like corn and soybeans. Thus, we could not disentangle the separate effects of comparison group, pesticide type, and crop type. In additional sensitivity analyses, we also developed separate models for atrazine and chlorpyrifos.

For residential use, we calculated an overall summary of the ratio in dust pesticide concentrations in homes reporting various home, garden, and yard pest treatments (high use group) versus those not reporting a given treatment (reference group). We also developed separate models for each active ingredient-pest treatment probability category, which were evaluated overall, using only the largest study (Deziel et al. 2015a), and using all studies but the largest. In addition, we evaluated the effect of the active ingredient probability category separately for herbicides and insecticides. The data stratified by probability category were too sparse to develop pesticide-specific models.

## Results

### Agricultural Drift Pathway

**Figure 1:**
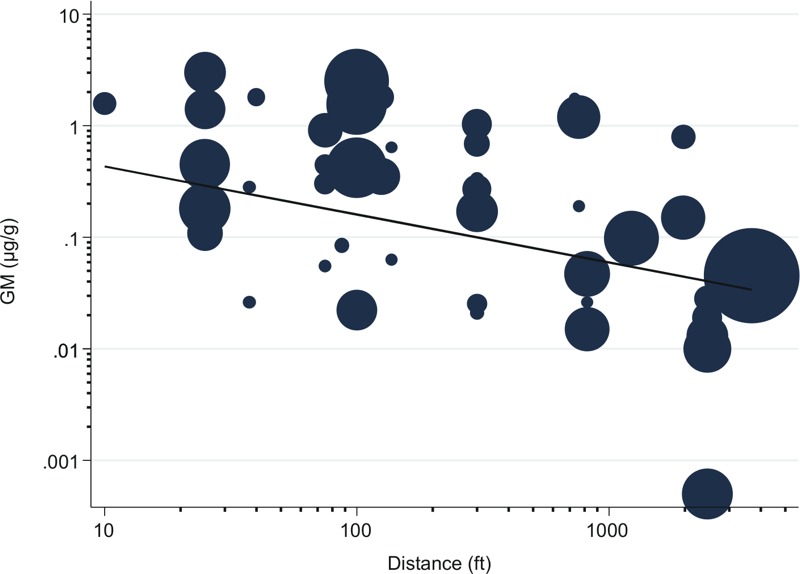
The GMs of concentrations of pesticide house dust decreased logarithmically with distance between home and treated fields. Solid line indicates predicted association from meta-regression models. Predicted GM at a given distance *d* in ft = *d* ^β^ ^slope^ *e* ^(^^β^ *^intercept^* ^)^ = *d* ^–0.43^ *e* ^0.15^. Circles indicate Distance/Pesticide/Paper-specific GMs, with circle width = (*logGSD*)*^2^*/*N_samples_*.

We identified seven studies reporting concentrations in house dust of multiple pesticide active ingredients in homes at varying distances from fields (Table 1) from which we extracted 52 sets of estimates. The reported distances ranged from 10 to 3,690 ft (3 to 1,125 m) with 25th, 50th, and 75th percentiles of 75 ft (23 m), 300 ft (91 m), and 820 ft (250 m), respectively. GSDs ranged from 1.4 to 10. Overall, house dust pesticide concentrations decreased sharply and non-linearly with increasing house distance from treated fields that was linear on a log-log scale, as shown in Figure 1. The model predicted GMs that were 64% lower in homes of 820 ft compared to 75 ft [the interquartile range (IQR)] and 35% lower in homes of 820 ft compared to 300 ft (75th percentile and median). The magnitude of decrease varied by pesticide type, with a 78% decrease in predicted GMs across the IQR for herbicides and fungicides and 51% across the IQR for insecticides (Table 2; see also Figure S1). These magnitudes of decreases were statistically different (*p*-value = 0.049) in a model that included all data and an interaction term for pesticide type and ln-distance. The magnitude of decline for chlorpyrifos mirrored that of all insecticides (50% across the IQR).

**Table 1:** Geometric means (GMs) of agricultural drift of dust pesticide concentrations in agricultural homes at varying distances from fields.

Author and year, state/pesticide	Pesticide type	Distance (ft)^*a*^	*N*_samples_	GM (μg/g)	GSD	Statistic ID	Pesticide-Paper ID
Fenske et al. 2002, Washington
Chlorpyrifos	Insecticide	100	46	0.42	2.30	11	15
Chlorpyrifos	Insecticide	300	15	0.17	2.05	12	15
Chlorpyrifos	Insecticide	25	33	0.45	2.35	15	15
Chlorpyrifos	Insecticide	125	13	0.35	2.10	16	15
Chlorpyrifos	Insecticide	760	4	0.19	4.40	17	15
Chlorpyrifos	Insecticide	1,980	11	0.15	1.75	18	15
Ethyl parathion	Insecticide	100	46	0.022	3.56	13	7
Ethyl parathion	Insecticide	300	15	0.025	4.56	14	7
Golla et al. 2012, Iowa
Atrazine-non-planting season^*b*^	Herbicide	37.5	10	0.026	8.5	50	10
Atrazine-non-planting season^*b*^	Herbicide	137.5	10	0.063	10.1	51	10
Atrazine-non-planting season^*b*^	Herbicide	300	11	0.021	7	52	10
Atrazine-planting season^*b*^	Herbicide	37.5	10	0.28	7.5	47	10
Atrazine-planting season^*b*^	Herbicide	137.5	10	0.64	10	48	10
Atrazine-planting season^*b*^	Herbicide	300	11	0.33	6.9	49	10
Gunier et al. 2011, California
Carbaryl	Insecticide	820	19	0.044	9	33	3
Carbaryl	Insecticide	2,460	70	0.015	9	34	3
Chlorpyrifos	Insecticide	820	68	0.047	4	35	16
Chlorpyrifos	Insecticide	2,460	21	0.028	4	36	16
Chlorthal	Herbicide	820	4	0.026	4	37	4
Chlorthal	Herbicide	2,460	85	0.0005	4	38	4
Diazinon	Insecticide	820	29	0.018	7	39	5
Diazinon	Insecticide	2,460	60	0.019	7	40	5
Iprodione	Fungicide	820	42	0.015	3	41	6
Iprodione	Fungicide	2,460	47	0.01	3	42	6
Phosmet	Insecticide	820	31	0.016	4	43	18
Phosmet	Insecticide	2,460	58	0.013	4	44	18
Simazine	Herbicide	820	43	0.041	5	45	8
Simazine	Herbicide	2,460	46	0.014	5	46	8
Lu et al. 2000, Washington
Azinphos methyl	Insecticide	100	45	1.6	2.30	5	12
Azinphos methyl	Insecticide	300	15	0.69	3.10	6	12
Dimethyl organophosphates	Insecticide	25	35	3	2.94	1	1
Dimethyl organophosphates	Insecticide	125	12	1.8	2.99	2	1
Dimethyl organophosphates	Insecticide	760	4	1.2	1.41	3	1
Dimethyl organophosphates	Insecticide	1,980	11	0.8	2.87	4	1
Dimethyl organophosphates	Insecticide	100	45	2.5	2.18	9	1
Dimethyl organophosphates	Insecticide	300	15	1.01	2.70	10	1
Phosmet	Insecticide	100	45	0.51	3.74	7	19
Phosmet	Insecticide	300	15	0.27	2.75	8	19
McCauley et al. 2001, Oregon
Azinphos methyl	Insecticide	10	6	1.6	2.3	29	13
Azinphos methyl	Insecticide	40	7	1.8	3.2	30	13
Azinphos methyl	Insecticide	87.5	5	0.085	3.5	31	13
Azinphos methyl	Insecticide	735	4	1.8	4.1	32	13
Simcox et al. 1995, Washington
Azinphos methyl	Insecticide	25	48	1.4	3.66	21	11
Azinphos methyl	Insecticide	75	15	0.91	2.38	22	11
Chlorpyrifos	Insecticide	25	48	0.18	2.74	25	14
Chlorpyrifos	Insecticide	75	15	0.30	3.96	26	14
Ethyl parathion	Insecticide	25	48	0.11	4.40	27	2
Ethyl parathion	Insecticide	75	15	0.055	12.7	28	2
Phosmet	Insecticide	25	48	0.35	10.1	23	17
Phosmet	Insecticide	75	15	0.45	4.40	24	17
Ward et al. 2006, Iowa
10 Herbicides	Herbicide	1,230	82	0.098	3.5	19	9
10 Herbicides	Herbicide	3,690	79	0.045	2.0	20	9
Note: GM, geometric mean; GSD, geometric standard deviation; Pesticide-Paper ID is a unique identifier assigned to each pesticide active ingredient-publication combination; Statistic ID is a unique identifier assigned to each GM. These IDs were used in the mixed-effects modeling [see “Appendix 1. Statistical (SAS) Code for Meta-Regression Models for Pesticide Exposure Pathways” in Supplemental Material]. ^***a***^Distance categories were assigned the midpoint of the category. ^***b***^Golla et al. (2012) measured atrazine at homes of varying distances to fields across two seasons.							

**Table 2:** Agricultural drift: model parameters for predicting the GMs of dust pesticide concentrations in agricultural homes at varying distances from fields.

Model	β (SE)	Exp(β) (95% CI)	Between-result variance, in ln μg/g (SE)	Between-pesticide/paper variance, in ln μg/g (SE)
All summary measures (*n* = 52)	—	—	0.80 (0.21)	1.56 (0.66)
Intercept	0.15 (0.72)	1.2 (0.28, 4.8)	—	—
Ln(distance in feet)	–0.43 (0.11)	0.65 (0.52, 0.81)	—	—
Herbicides/fungicides (*n* = 14)	—	—	2.0 (0.77)	Not estimated
Intercept	0.68 (1.7)	2.0 (0.07, 52)	—	—
Ln(distance in feet)	–0.64 (0.26)	0.53 (0.32, 0.88)	—	—
Insecticides (*n* = 38)	—	—	0.33 (0.10)	1.79 (0.75)
Intercept	–0.22 (0.59)	0.80 (0.25, 2.6)	—	
Ln(distance in feet)	–0.30 (0.09)	0.74 (0.62, 0.88)	—	—
Chlorpyrifos (*n* = 10)	—	—	0.11 (0.06)	~ 0
Intercept	–0.43 (0.55)	0.65 (0.22, 1.9)	—	—
Ln(distance in feet)	–0.29 (0.08)	0.75 (0.64, 0.87)	—	—
Note: —, data not available; CI, confidence interval; GM, geometric mean; SE, standard error. Predicted GM at a given distance = *d*^β^^*slope*^ exp(β_*intercept*_) (Equation 13). Percent (%) change between distances *d*_1_ and *d*_2_ = [1 – (*d*_2_/*d*_1_)^β^^*slope*^] × 100 (Equation 14).				

### Para-Occupational Pathway

We identified five studies reporting pesticide concentrations in house dust that could be used to quantify the mean difference between homes of farmers with high vs. low pesticide use (Table 3). From these studies we derived 15 estimates of the ratio of GMs (GM Ratio) for homes of farmers in the high use versus reference group. The GM ratios varied from 0.57 to 31 and the variances of the ln-ratios varied across three orders of magnitude.

**Table 3:** Para-occupational exposure: Ratios of GMs of dust pesticide concentrations between measurements taken in homes of farmers with high pesticide use compared to those with low pesticide use.

Author and year, state/pesticide	Pesticide type	Exposure comparison groups	Low-pesticide use group (reference)				High-pesticide use group				Ratios			Statistic ID
			*N*_homes_	*N*_samples_^*a*^	GM (μg/g)	GSD	*N*_homes_	*N*_samples_^*a*^	GM (μg/g)	GSD	GM ratio (high/ref)	ln-ratio	Variance of ln-ratio	
Curwin et al. 2005, Iowa
2,4-D	Herbicide	Farmers who applied 2,4-D < 30 vs. ≥ 30 days before dust sampling	3	3.8	0.340	2.7	2	2.3	1.7	4.0	5.00	1.6	1.1	15
Atrazine	Herbicide	Farmers who applied active ingredient of interest < 7 vs. ≥ 7 days before dust sampling	≤ 20^*b*^	16.1	0.016	11	≤ 20^*b*^	16	0.17	11	11	2.4	0.71	11
Chlorpyrifos	Insecticide		≤ 20^*b*^	50.0	0.010	13	2	1.2	0.07	2.1	1.9	0.63	0.68	13
Glyphosate	Herbicide		≤ 5^*b*^	8.2	0.92	2.1	≤ 5^*b*^	5.9	1.1	2.4	1.2	0.18	0.20	14
Metolachlor	Herbicide		≤ 20^*b*^	50.0	0.010	13	≤ 20^*b*^	11	0.31	20	31	3.4	0.94	12
Fenske et al. 2002, Washington
Chlorpyrifos	Insecticide	Farmers who apply vs. do not apply pesticides	12	12	0.225	1.8	49	49	0.38	2.4	1.7	0.52	0.046	1
Ethyl parathion	Insecticide		12	12	0.005	5.4	49	49	0.03	3.9	5.8	1.8	0.27	2
Golla et al. 2012, Iowa
Atrazine	Herbicide	Farmers who applied atrazine ≥ 2 vs. < 2 days per season	16	17.8	0.241	6.7	15	17	0.65	8.5	2.7	0.99	0.48	3
Lu et al. 2000, Washington
Azinphos methyl	Insecticide	Farmers who apply vs. do not apply pesticides	13	13	1.03	2.3	49	49	1.4	2.5	1.3	0.29	0.071	4
Dimethyl OPs	Insecticide		13	13	1.1	2.3	49	49	2.4	2.3	2.1	0.73	0.066	6
Phosmet	Insecticide		13	13	0.11	1.9	49	49	0.54	3.6	4.8	1.6	0.065	5
Simcox et al. 1995, Washington
Azinphosmethyl	Insecticide	Farmers who apply vs. do not apply pesticides	20	20	1.4	1.2	28	28	1.1	1.0	0.74	–0.30	0.002	7
Chlorpyrifos	Insecticide		20	20	0.30	0.54	28	28	0.17	0.41	0.57	–0.56	0.047	9
Ethyl parathion	Insecticide		20	20	0.043	0.21	28	28	0.05	0.23	1.23	0.21	0.20	10
Phosmet	Insecticide		20	20	0.30	0.54	28	28	0.32	0.57	1.09	0.083	0.030	8
Note: 2,4-D, 2,4-dichlorophenoxyacetic acid; GM, geometric mean; GSD, geometric standard deviation; OP, organophosphate; Ref, reference; Statistic ID is a unique identifier assigned to each GM ratio: This ID was used in the mixed-effects modeling [see “Appendix 1. Statistical (SAS) Code for Meta-Regression Models for Pesticide Exposure Pathways” in Supplemental Material]. ^***a***^For studies with repeated measures at a home (Curwin et al. 2005; Golla et al. 2012), the *N*_*samples*_ refers to the effective sample size (see Equations 7 and 8). ^***b***^Used median of category in design effect calculations.														

Overall, in a meta-regression model, we found that dust pesticide concentrations were 2.3 times higher [95% confidence interval (CI): 1.5, 3.3] in homes of farmers with high pesticide use versus the reference group (Table 4). Sensitivity analyses indicated higher ratios in studies of farmers who applied specific pesticides in specific time windows or frequencies (Ratio: 3.8, 95% CI: 1.6, 9.2) than in studies with more general comparisons between farmers who applied or did not apply pesticides (Ratio: 2.0, 95% CI: 1.3, 3.0). It is not clear if these differences were related to differences between the comparison group type or pesticide type. However, these differences between the ratios were not statistically significant in a model that included all data and that incorporated pesticide type as an explanatory variable (*p*-value = 0.14). Additionally, the ratios were higher for atrazine (Ratio: 4.7, 95% CI: 1.6, 13) than for chlorpyrifos (Ratio: 1.6, 95% CI: 1.1, 2.3) (*p*-value for comparison = 0.06); however, these comparisons were based on small numbers. The between-statistic variances estimated by these models were not statistically significantly different from zero.

**Table 4:** Para-occupational exposure: Predicted ratios of dust pesticide concentrations of homes of farmers with high versus low pesticide use based on meta-regression models.

Model	Predicted ratio for high vs. low use^*a*^ (95% CI)	Between-result variance, in log-μg/g (SE)
All summary measures (*n* = 15)	2.3 (1.5, 3.3)	0.26 (0.23)
Farmers who apply vs. do not apply pesticides^*b*^ (*n* = 9, all insecticides)	2.0 (1.3, 3.0)	0.21 (0.19)
Farmers who applied specific pesticides in specific time windows or at specific frequencies^*c*^ (*n* = 6, all herbicides)	3.8 (1.6, 9.2)	0.61 (0.68)
Atrazine (*n* = 2)	4.7 (1.6, 13)	~ 0
Chlorpyrifos (*n* = 3)	1.6 (1.1, 2.3)	~ 0
Note: CI, confidence interval; SE, standard error. ^***a***^Ratios of dust pesticide concentrations of homes of farmers with high versus low pesticide use based on meta-regression models. Calculated as the exponentiated regression coefficient for the intercept from the meta-regression model. ^***b***^Excludes Golla et al. (2012) and Curwin et al. (2005). ^***c***^Includes two studies: Curwin et al. (2005), which compared applications < 7 vs. ≥ 7 and < 30 vs. ≥ 30 days and Golla et al. (2012), which compared application ≥ 2 vs. < 2 days per season.		

### Residential Use Pathway

We identified five studies reporting pesticide concentrations in house dust in agricultural homes that were treated or not treated (i.e., high use vs. reference) for various insects and weeds in the home, garden, or yard (Table 5). From these studies we derived 88 estimates of the ratios of GMs between treated vs. non-treated homes. The GM ratios varied from 0.22 to 6.8. Overall, dust pesticide concentrations were 1.3 times higher (95% CI: 1.1, 1.4) in households that treated vs. did not treat their homes, gardens, or yards for insect or weeds (Table 6). The magnitude of the contribution increased with the probability of use of an active ingredient for the specific pest treatment. For probability categories 0%, 1–19%, and ≥ 20%, respectively, the dust pesticide concentrations were 1.0 (95% CI: 0.8, 1.3), 1.3 (95% CI: 1.1, 1.4), and 1.5 (95% CI: 1.2, 1.9) times higher in households that treated vs. did not treat. The magnitude of the effect by probability category was somewhat larger when Deziel et al. 2015a was excluded (Table 6). However, this effect was only statistically different at the 1–19% probability category in models that included all data and a fixed-effect term for data source (1 = Deziel et al. 2015a; 0 = all other studies; *p*-values of 0.5, 0.002 and 0.4 for probability categories 0%, 1–19%, and ≥ 20%, respectively; not shown). Stratified analyses also showed some differences in the magnitude of effect between herbicides and insecticides for the 1–19% probability category, but not the 0% and ≥ 20% categories (Table 6); this difference was not statistically significant (*p*-value = 0.2).

**Table 5:** Residential use exposure: Ratios of GMs of dust pesticide concentrations between measurements taken in agricultural homes that were treated (high use) versus not treated (reference) for home, garden, or yard insects or weeds.

Author and year, state/pesticide	Pesticide type	Treatment type^*a*^	*N*_samples_, high use	*N*_samples_, reference	GM ratio (high/reference)^*b*^	ln-ratio	Variance ln-ratio	Probability	Statistic ID
Deziel et al. 2013, California
Carbaryl	Insecticide	Ants/flies/roaches	36	32	2.3	0.81	0.10	1–19%	1
Carbaryl	Insecticide	Professional outdoor	17	44	1.2	0.19	0.16	0%	2
Carbaryl	Insecticide	Professional outdoor or indoor	7	44	0.41	–0.89	0.27	0%	3
Chlorpyrifos	Insecticide	Bees/wasps/hornets	11	57	1.9	0.62	0.06	1–19%	4
Cyfluthrin	Insecticide	Professional outdoor	17	44	4.7	1.55	0.18	1–19%	5
Cyfluthrin	Insecticide	Professional outdoor or indoor	7	44	1.1	0.07	0.36	1–19%	6
Cypermethrin	Insecticide	Professional outdoor	17	44	3.3	1.20	0.18	≥ 20%	7
Cypermethrin	Insecticide	Professional outdoor or indoor	7	44	1.4	0.34	0.25	≥ 0%	8
Diazinon	Insecticide	Lawn/garden	38	30	1.7	0.55	0.07	≥ 20%	9
Diazinon	Insecticide	Professional outdoor	17	44	3.0	1.09	0.18	1–19%	10
Diazinon	Insecticide	Professional outdoor or indoor	7	44	0.70	–0.36	0.41	1–19%	11
Methoxychlor	Insecticide	Professional outdoor	17	44	1.6	0.44	0.31	0%	12
Methoxychlor	Insecticide	Professional outdoor or indoor	7	44	0.22	–1.51	0.56	0%	13
Permethrin	Insecticide	Professional outdoor	17	44	3.5	1.25	0.12	0%	14
Permethrin	Insecticide	Professional outdoor or indoor	7	44	1.3	0.25	0.08	1–19%	15
Gunier et al. 2011, California
Chlorpyrifos	Insecticide	Fleas/ticks	32	57	2.0	0.68	0.08	1–19%	16
Diazinon	Insecticide	Professional outdoor	89	89	2.6	0.94	0.23	1–19%	17
Phosmet	Insecticide	Professional outdoor	23	66	1.7	0.53	1.79	0%	18
Lu et al. 2000, Washington
DimethylOP	Insecticide	Fleas/ticks	16	84	0.33	–1.10	0.05	0%	19
DimethylOP	Insecticide	Garden insects	29	61	1.1	0.10	0.04	0%	20
DimethylOP	Insecticide	Lawn insects	31	69	1.4	0.37	0.03	1–19%	21
Golla et al. 2012, Iowa
Atrazine	Herbicide	Lawn	8	23	1.6	0.47	4.68	1–19%	22
Deziel et al. 2015a, California
2,4-D	Herbicide	Professional weeds	57	444	0.54	–0.62	0.05	≥ 20%	23
2,4-D	Herbicide	Weeds	269	304	2.8	1.03	0.02	≥ 20%	24
Carbaryl	Insecticide	Ants/cockroach	409	162	0.72	–0.33	0.07	1–19%	25
Carbaryl	Insecticide	Carpenter ants/termites	33	153	1.4	0.34	0.23	0%	26
Carbaryl	Insecticide	Fleas/ticks in the home	59	516	1.9	0.64	0.14	1–19%	27
Carbaryl	Insecticide	Fleas/ticks on pets	147	428	0.94	–0.06	0.07	1–19%	28
Carbaryl	Insecticide	Flying insects	162	409	0.88	–0.13	0.07	1–19%	29
Carbaryl	Insecticide	Lawn/garden insects	170	401	1.3	0.26	0.06	1–19%	30
Carbaryl	Insecticide	Professional indoor	66	437	1.5	0.41	0.18	0%	31
Carbaryl	Insecticide	Professional outdoor	139	355	0.71	–0.34	0.12	0%	32
Chlorpyrifos	Insecticide	Ants/cockroach	409	162	0.99	–0.01	0.02	1–19%	33
Chlorpyrifos	Insecticide	Carpenter ants/termites	33	153	1.7	0.53	0.07	≥ 20%	34
Chlorpyrifos	Insecticide	Fleas/ticks in the home	59	516	0.99	–0.01	0.04	1–19%	35
Chlorpyrifos	Insecticide	Fleas/ticks on pets	147	428	0.99	–0.01	0.02	0%	36
Chlorpyrifos	Insecticide	Flying insects	162	409	0.80	–0.22	0.02	1–19%	37
Chlorpyrifos	Insecticide	Lawn/garden insects	170	401	1.7	0.53	0.02	≥ 20%	38
Chlorpyrifos	Insecticide	Professional indoor	66	437	1.5	0.41	0.05	1–19%	39
Chlorpyrifos	Insecticide	Professional outdoor	139	355	0.85	–0.16	0.03	≥ 20%	40
Chlorthal	Herbicide	Professional weeds	57	444	0.72	–0.33	0.09	0%	41
Chlorthal	Herbicide	Weeds	269	304	1.3	0.26	0.04	1–19%	42
Cyfluthrin	Insecticide	Ants/cockroach	409	162	0.70	–0.36	0.13	1–19%	43
Cyfluthrin	Insecticide	Carpenter ants/termites	33	153	1.2	0.18	0.36	0%	44
Cyfluthrin	Insecticide	Fleas/ticks in the home	59	516	1.5	0.41	0.23	1–19%	45
Cyfluthrin	Insecticide	Fleas/ticks on pets	147	428	0.95	–0.05	0.13	0%	46
Cyfluthrin	Insecticide	Flying insects	162	409	1.1	0.10	0.13	1–19%	47
Cyfluthrin	Insecticide	Lawn/garden insects	170	401	2.3	0.83	0.11	0%	48
Cyfluthrin	Insecticide	Professional indoor	66	437	4.0	1.39	0.26	1–19%	49
Cyfluthrin	Insecticide	Professional outdoor	139	355	6.8	1.92	0.17	1–19%	50
Cypermethrin	Insecticide	Ants/cockroach	409	162	2.5	0.92	0.07	1–19%	51
Cypermethrin	Insecticide	Carpenter ants/termites	33	153	0.91	–0.09	0.22	1–19%	52
Cypermethrin	Insecticide	Fleas/ticks in the home	59	516	1.9	0.64	0.13	0%	53
Cypermethrin	Insecticide	Fleas/ticks on pets	147	428	0.65	–0.43	0.07	0%	54
Cypermethrin	Insecticide	Flying insects	162	409	1.7	0.53	0.06	1–19%	55
Cypermethrin	Insecticide	Lawn/garden insects	170	401	1.2	0.18	0.06	0%	56
Cypermethrin	Insecticide	Professional indoor	66	437	0.91	–0.09	0.16	≥ 20%	57
Cypermethrin	Insecticide	Professional outdoor	139	355	2.3	0.83	0.09	0%	58
Diazinon	Insecticide	Ants/cockroach	409	162	1.00	0.00	0.03	1–19%	59
Diazinon	Insecticide	Carpenter ants/termites	33	153	1.4	0.34	0.09	0%	60
Diazinon	Insecticide	Fleas/ticks in the home	59	516	1.5	0.41	0.06	1–19%	61
Diazinon	Insecticide	Fleas/ticks on pets	147	428	0.87	–0.14	0.03	0%	62
Diazinon	Insecticide	Flying insects	162	409	0.92	–0.08	0.03	1–19%	63
Diazinon	Insecticide	Lawn/garden insects	170	401	1.5	0.41	0.03	≥ 20%	64
Diazinon	Insecticide	Professional indoor	66	437	1.5	0.41	0.07	1–19%	65
Diazinon	Insecticide	Professional outdoor	139	355	1.5	0.41	0.05	1–19%	66
Dicamba	Herbicide	Professional weeds	57	444	0.90	–0.11	0.06	≥ 20%	67
Dicamba	Herbicide	Weeds	269	304	1.9	0.64	0.02	≥ 20%	68
Mecoprop	Herbicide	Professional weeds	57	444	0.60	–0.51	0.06	1–19%	69
Mecoprop	Herbicide	Weeds	269	304	2.2	0.79	0.03	≥ 20%	70
Permethrin	Insecticide	Ants/cockroach	409	162	1.3	0.26	0.03	1–19%	71
Permethrin	Insecticide	Carpenter ants/termites	33	153	0.91	–0.09	0.10	1–19%	72
Permethrin	Insecticide	Fleas/ticks in the home	59	516	2.3	0.83	0.06	≥ 20%	73
Permethrin	Insecticide	Fleas/ticks on pets	147	428	1.2	0.18	0.03	1–19%	74
Permethrin	Insecticide	Flying insects	162	409	1.6	0.47	0.03	≥ 20%	75
Permethrin	Insecticide	Lawn/garden insects	170	401	0.74	–0.30	0.02	0%	76
Permethrin	Insecticide	Professional indoor	66	437	1.6	0.47	0.08	1–19%	77
Permethrin	Insecticide	Professional outdoor	139	355	0.97	–0.03	0.05	1–19%	78
Propoxur	Insecticide	Ants/cockroach	409	162	1.3	0.26	0.03	1–19%	79
Propoxur	Insecticide	Carpenter ants/termites	33	153	0.77	–0.26	0.09	0%	80
Propoxur	Insecticide	Fleas/ticks in the home	59	516	1.4	0.34	0.05	1–19%	81
Propoxur	Insecticide	Fleas/ticks on pets	147	428	1.3	0.26	0.03	1–19%	82
Propoxur	Insecticide	Flying insects	162	409	0.88	–0.13	0.03	1–19%	83
Propoxur	Insecticide	Lawn/garden insects	170	401	0.88	–0.13	0.03	0%	84
Propoxur	Insecticide	Professional indoor	66	437	0.89	–0.12	0.07	0%	85
Propoxur	Insecticide	Professional outdoor	139	355	0.80	–0.22	0.04	0%	86
Simazine	Herbicide	Professional weeds	57	444	1.1	0.10	0.03	1–19%	87
Simazine	Herbicide	Weeds	269	304	1.1	0.10	0.01	0%	88
Note: GM, geometric mean; Statistic ID is a unique identifier assigned to each GM ratio: It was used in the mixed-effects modeling [see “Appendix 1. Statistical (SAS) Code for Meta-Regression Models for Pesticide Exposure Pathways” in Supplemental Material]. ^***a***^Professional describes a pest treatment applied by a commercial applicator, while the other treatments describe those done by a resident. ^***b***^Except for Lu et al. (2000) and Golla et al. (2012), the ratio was obtained from regression models and as a result the GMs for reference and comparison groups were not available.									

**Table 6:** Residential use: Predicted ratio in GM dust pesticide concentrations in treated versus untreated agricultural homes.

Model	Predicted ratio for treated vs. untreated homes (95% CI)^*a*^	Between-statistic variance, in log-μg/g (SE)
All summary measures (*n* = 88)	1.3 (1.1, 1.4)	0.15 (0.04)
Probability active ingredient used in treatment type^*b*^
All studies
Probability: 0% (*n* = 28)	1.0 (0.8, 1.2)	0.15 (0.07)
Probability: 1–19% (*n* = 45)	1.3 (1.1, 1.4)	0.08 (0.04)
Probability: ≥ 20% (*n* = 15)	1.5 (1.2, 1.9)	0.16 (0.08)
Deziel et al. 2015a^*c*^
Probability: 0% (*n* = 20)	1.0 (0.9, 1.2)	0.03 (0.04)
Probability: 1–19% (*n* = 34)	1.2 (1.1, 1.3)	0.06 (0.03)
Probability: ≥ 20% (*n* = 12)	1.5 (1.1, 1.9)	0.17 (0.09)
Excluding Deziel et al. 2015a^*c*^
Probability: 0% (*n* = 8)	0.9 (0.5, 1.6)	0.56 (0.83)
Probability: 1–19% (*n* = 11)	1.8 (1.5, 2.2)	0.01 (0.05)
Probability: ≥ 20% (*n* = 3)	2.0 (1.3, 2.9)	(single study)
Herbicides
Probability: 0% (*n* = 2)	1.0 (0.9, 1.3)	(single study)
Probability: 1–19% (*n* = 4)	1.0 (0.7, 1.4)	0.04 (0.08)
Probability: ≥ 20% (*n* = 5)	1.5 (0.9, 2.5)	0.33 (0.23)
Insecticides
Probability: 0% (*n* = 26)	1.0 (0.8, 1.3)	0.17 (0.08)
Probability: 1–19% (*n* = 41)	1.3 (1.2, 1.5)	0.08 (0.04)
Probability: ≥ 20% (*n* = 10)	1.5 (1.3, 1.9)	0.05 (0.05)
^***a***^Ratios of dust pesticide concentrations of homes treated versus not treated for home, garden, and yard pests based on meta-regression models. Calculated as the exponentiated regression coefficient for the intercept from the meta-regression model. ^***b***^Probability was assigned to each active ingredient-pest treatment relationship, based on the likelihood the active ingredient was used for a specific pest treatment based on the National Cancer Institute Pesticide Exposure Matrix (http://dceg.cancer.gov/tools/design/pesticide) (Colt et al. 2007). ^***c***^Deziel et al. (2015a) was analyzed separately because it was the largest study containing 74 of the 88 summary statistics.		

## Discussion

To our knowledge, this is the first use of meta-regression models to summarize environmental pesticide concentrations reported in the published literature. This approach allowed us to estimate the average ratios in pesticide concentration in house dust from various pathways across multiple studies while accounting for both the study size and concentration variability. Overall, pesticide concentrations in house dust decreased rapidly with increasing distance, with predicted GMs decreasing 64% across the IQR of the published data. Pesticide concentrations in dust were also 2.3 times higher (95% CI: 1.5, 3.3) in homes where a resident had high versus low agricultural use of pesticides and 1.3 times higher (95% CI; 1.1, 1.4) in homes where pesticides were used in the home, garden, or yard versus not used for specific pests. These findings provide data-driven weights, with confidence intervals, that could be used in future exposure assessment efforts in epidemiologic studies. In addition, this study provides a framework for applying meta-regression models to analyze published data for other exposures and exposure determinants of interest.

The contribution of the residential use pathway increased with increasing probability of the pesticide treatment type including the active ingredient, but there was little evidence for subgroup differences in the other two pathways. Sensitivity analyses suggested that the magnitude of the contribution of each pathway may differ by pesticide type or active ingredient. At this time, we have insufficient evidence to confirm these differences, as sample sizes were generally small. For example, agricultural drift might be influenced by pesticide type or active ingredient (e.g., due to differences in the volatility of active ingredients), crop type, meteorology, and pesticide application method (Damalas and Eleftherohorinos 2011; Ward et al. 2006). However, in our comparisons, the strong correlations among potential explanatory variables prevented us from disentangling whether the observed differences were attributable to any of those factors, and important differences may have been missed because of sparse data. Because of our transparent approach, our results can be updated as more data becomes available.

Using a mixed-effects model framework provided an opportunity to systematically account for both the within-study and between-study variability for specific pesticides and the power of the study based on the number of measurements. However, differences may still have been masked. For instance, in the agricultural drift model, there is potential for aggregation bias because we evaluated only an overall trend, rather than pesticide/study-specific trends. Preliminary models that incorporated a random slope did not detect pesticide/study-specific trends, but differences may have been missed due to computational limitations and sparse data. Visual inspection showed that most pesticide/study-specific trends paralleled the overall trend (see Figure S2); for these the random intercept would be sufficient to capture the offset in the intercept. However, we may have missed important differences for the small number of trends that did not parallel the overall trend. This challenge was also encountered in a previous meta-regression analysis of occupational lead data, where industry-specific temporal trends were unable to capture differences related to variability in the jobs that were monitored (Koh et al. 2014).

These models estimated an average effect that provides an estimate of the median change in dust pesticide concentration between distances (drift) or comparison groups (para-occupational, residential use pathways). In epidemiologic studies, comparing arithmetic means may be of greater interest. The arithmetic mean (AM) can be approximated using the equation AM = exp(β + 0.5 * SE^2^), where β and SE are the model parameter and its standard error before exponentiating the terms. Here, for the three pathways, the AM and GM were identical at one decimal place (not shown). Estimating the ratio of the AMs between comparison groups, that is, AM*_high_*/AM*_reference_* instead of GM*_high_*/GM*_reference_*, is more challenging, because the log-normal statistical properties of the former’s exposure distribution are more difficult to obtain and incorporate into statistical models. More flexible modeling approaches, such as Bayesian approaches that can specify different exposure distributions for each parameter, may address this challenge.

### Limitations

There were several additional limitations to these analyses related to the coverage of the data and use of surrogates to represent these three exposure pathways. First, the magnitude of these differences may be overestimated due to publication bias because studies that observed no association between pesticide house dust levels and a particular pathway often did not report summary statistics or regression coefficients and could not be included here. Publication bias may account for differences in the contribution of the residential use pathway between the Deziel et al. (2015a) study and all other papers. The Deziel et al. (2015a) study included 74 of the 88 statistics and reported all possible comparisons between multiple pesticides and pest treatments, whereas other papers evaluating several pesticides generally reported only statistically significant findings. For the agricultural drift pathway, several studies stated that they did not observe an association between dust pesticide concentrations and distance from home to treated fields without providing the underlying summary statistics (Coronado et al. 2011; Curwin et al. 2005; McCauley et al. 2003). However, in these studies, the homes tended to be located very close to the fields, limiting the variability in distance categories. Second, as described above, the data were generally too sparse to identify whether differences in pesticide house dust concentrations varied by subgroups (e.g., pesticide type, crop type, application method, geographic location, or time period) and important distinctions may have been missed. Third, we used exposure surrogates to create our comparison groups; the exposure pathways may be better characterized with other metrics. For instance, compared to self-reported distance to treated fields, agricultural drift may be better captured using geographic information systems approaches that use satellite images, crop maps, historical farm records, and state pesticide use reporting databases to better classify exposure according to crop acreage or quantity of active ingredients applied near residences (Fenske 2005; Gunier et al. 2011; Harnly et al. 2009; Jones et al. 2014; Ritz and Rull 2008; Ward et al. 2000); Fourth, most of the studies were based in the northwestern United States (Washington and Oregon) and Iowa, and thus the results may not be generalizable to populations in other geographic regions. Lastly, the lack of reporting of active ingredient-specific information in the published studies of the residential use treatments, and the resulting use of group-level probability-based weights from the NCI pesticide exposure matrix, introduces uncertainty in the quantification of the contribution of the residential use pathway. This pesticide exposure matrix was last updated with market and usage data from the year 2000 and may have limited relevance for informing residential use of certain pesticides subsequent to that year.

There were also several limitations to the abstracted data and the modeling framework. First, it is difficult to disentangle the independent contribution of each pathway. Although we abstracted data that accounted for the other potential pathways through adjustment in multivariable regression models or stratification wherever possible, the estimates of the contributions of each pathway may be confounded by other pathways. Second, development of the richest data source possible required approximations, with varying errors, when GMs and GSDs were not directly reported. For example, we assumed the median was approximately equivalent to the GM. In addition, we visually extracted medians from graphs in four of the seven studies of agricultural drift, which introduced imprecision in the estimates. Similarly, based on visual inspection of the data, we assumed a lognormal distribution for both the dust pesticide concentrations and the ratios. Deviations from this assumption could affect the point estimates, *p*-values and confidence intervals. As a result, we presented results only to 2 significant figures and we use confidence intervals and *p*-values as guides and not definitive measures of scientific significance.

These findings provide insight into the contributions of these exposure pathways to the indoor dust pesticide concentrations; however, the impact of these differences to the pesticide exposure of adults remains uncertain because individual behaviors and characteristics also influence the amount of pesticide exposure and absorption (Hoppin et al. 2006). Pesticide concentrations in air, food, water, and biological specimens may also be used to represent adult exposure and dose. However, our prior review found that evaluations in media other than dust were rare, often had low detection rates, and for biomarkers represented only very recent exposure (Deziel et al. 2015b); as a result, these metrics were not included in these analyses. Previous studies that have compared concentrations or loadings of pesticides in bulk dust or wipes with concentrations of pesticide biomarkers in adults have observed weak to moderate correlations or associations (Thompson et al. 2014; Curwin et al. 2007; Arbuckle et al. 2006). However, making these comparisons is challenging because the varying media reflect different exposure windows, with dust samples representing a cumulative time window representing weeks, months, or years, and biomarkers often representing exposure in the hours to days prior to sample collection (Barr et al. 2006; Bouvier et al. 2005; Morgan et al. 2008). Future research with repeated biological measures would advance our understanding of the predictive value of pesticide house dust measurements for long-term exposures in adults. In addition, the framework used here can be expanded to other sample media as more data becomes available.

## Conclusion

We used a novel application of meta-analysis to published pesticide exposure data to quantify the relative difference in dust pesticide concentrations in relation to surrogates representing three pesticide use and transport exposure pathways in agricultural populations. Our analyses found that homes near treated fields, homes of farmers who applied pesticides more frequently or recently, and homes of those who applied pesticides around the home, garden, and yard, had quantifiably higher pesticide concentrations in the dust compared to their reference groups. These results can inform the development of data-driven environmental exposure categorizations for epidemiologic studies. Our transparent meta-regression models can be updated when new data are available or further restricted or expanded based on the population of interest. Additionally, the framework developed for these analyses can be applied to other published exposure data.

## Supplemental Material

(95 KB) PDF

(5 KB) ZIP

## References

[r1] Aitchison J, Brown JAC (1963). *The Lognormal Distribution:* with Special Reference to Its Uses in Economics..

[r2] Arbuckle TE, Bruce D, Ritter L, Hall JC (2006). Indirect sources of herbicide exposure for families on Ontario farms.. J Expo Sci Environ Epidemiol.

[r3] Bain R Cronk R Wright J Yang H Slaymaker T Bartram J 2014 Fecal contamination of drinking-water in low- and middle-income countries: a systematic review and meta-analysis. PLoS Med 11 5 e1001644, doi:10.1371/journal.pmed.1001644 24800926 PMC4011876

[r4] Barr DB Thomas K Curwin B Landsittel D Raymer J Lu C 2006 Biomonitoring of exposure in farmworker studies. Environ Health Perspect 114 936 942, doi:10.1289/ehp.8527 16759998 PMC1480485

[r5] Bouvier G, Seta N, Vigouroux-Villard A, Blanchard O, Momas I (2005). Insecticide urinary metabolites in nonoccupationally exposed populations.. J Toxicol Environ Health B Crit Rev.

[r6] Butte W, Heinzow B (2002). Pollutants in house dust as indicators of indoor contamination.. Rev Environ Contam Toxicol.

[r7] Colt JS Cyr MJ Zahm SH Tobias GS Hartge P 2007 Inferring past pesticide exposures: a matrix of individual active ingredients in home and garden pesticides used in past decades. Environ Health Perspect 115 248 254, doi:10.1289/ehp.9538 17384773 PMC1817710

[r8] Colt JS, Lubin J, Camann D, Davis S, Cerhan J, Severson RK (2004). Comparison of pesticide levels in carpet dust and self-reported pest treatment practices in four US sites.. J Expo Anal Environ Epidemiol.

[r9] Coronado GD, Holte S, Vigoren E, Griffith WC, Barr DB, Faustman E (2011). Organophosphate pesticide exposure and residential proximity to nearby fields: evidence for the drift pathway.. J Occup Environ Med.

[r10] Curl CL, Fenske RA, Kissel JC, Shirai JH, Moate TF, Griffith W (2002). Evaluation of take-home organophosphorus pesticide exposure among agricultural workers and their children.. Environ Health Perspect.

[r11] Curwin BD, Hein MJ, Sanderson WT, Nishioka MG, Reynolds SJ, Ward EM (2005). Pesticide contamination inside farm and nonfarm homes.. J Occup Environ Hyg.

[r12] Curwin BD, Hein MJ, Sanderson WT, Striley C, Heederik D, Kromhout H (2007). Urinary pesticide concentrations among children, mothers and fathers living in farm and non-farm households in Iowa.. Ann Occup Hyg.

[r13] Damalas CA, Eleftherohorinos IG (2011). Pesticide exposure, safety issues, and risk assessment indicators.. Int J Environ Res Public Health.

[r14] Deziel NC Colt JS Kent EE Gunier RB Reynolds P Booth B 2015a Associations between self-reported pest treatments and pesticide concentrations in carpet dust. Environ Health 14 27, doi:10.1186/s12940-015-0015-x 25889489 PMC4374193

[r15] Deziel NC Friesen MC Hoppin JA Hines CJ Thomas K Beane Freeman LE 2015b A review of nonoccupational pathways for pesticide exposure in women living in agricultural areas. Environ Health Perspect 123 515 524, doi:10.1289/ehp.1408273 25636067 PMC4455586

[r16] Deziel N Ward M Bell E Whitehead T Gunier R Friesen M 2013 Temporal variability of pesticide concentrations in homes and implications for attenuation bias in epidemiologic studies. Environ Health Perspect 121 565 571, doi:10.1289/ehp.1205811 23462689 PMC3672902

[r17] Fenske RA (2005). State-of-the-art measurement of agricultural pesticide exposures.. Scan J Work Environ Health.

[r18] Fenske RA, Lu C, Barr D, Needham L (2002). Children’s exposure to chlorpyrifos and parathion in an agricultural community in central Washington State.. Environ Health Perspect.

[r19] Golla V Curwin B Sanderson W Nishioka M 2012 Pesticide concentrations in vacuum dust from farm homes: variation between planting and nonplanting seasons. ISRN Public Health 2012 539397, doi:10.5402/2012/539397

[r20] Gunier RB Ward MH Airola M Bell EM Colt J Nishioka M 2011 Determinants of agricultural pesticide concentrations in carpet dust. Environ Health Perspect 119 970 976, doi:10.1289/ehp.1002532 21330232 PMC3222988

[r21] Harnly ME, Bradman A, Nishioka M, McKone TE, Smith D, McLaughlin R (2009). Pesticides in dust from homes in an agricultural area.. Environ Sci Technol.

[r22] Hein MJ, Waters MA, van Wijngaarden E, Deddens JA, Stewart PA (2008). Issues when modeling benzene, toluene, and xylene exposures using a literature database.. J Occ Environ Hyg.

[r23] Hewett P (2005). Equations for calculating exposure manage ment objectives. Technical Report No. 05–02.. http://www.easinc.co/wp-content/uploads/2016/03/TR-05-02-ContObj.pdf.

[r24] Hoppin JA Adgate JL Eberhart M Nishioka M Ryan PB 2006 Environmental exposure assessment of pesticides in farmworker homes. Environ Health Perspect 114 929 935, doi:10.1289/ehp.8530 16759997 PMC1480520

[r25] Jones RR Yu CL Nuckols JR Cerhan JR Airola M Ross JA 2014 Farm residence and lymphohematopoietic cancers in the Iowa Women’s Health Study. Environ Res 133 353 361, doi:10.1016/j.envres.2014.05.028 25038451 PMC4324553

[r26] Kish L (1965). *Survey Sampling*..

[r27] Koh DH, Nam JM, Graubard BI, Chen YC, Locke SJ, Friesen MC (2014). Evaluating temporal trends from occupational lead exposure data reported in the published literature using meta-regression.. Ann Occup Hyg.

[r28] Konstantopoulos S, Hedges LV (2004). Meta-analysis. In: *The SAGE Handbook of Quantitative Methodology for the Social Sciences*. Kaplan D, ed..

[r29] Lavoué J, Bégin D, Beaudry C, Gérin M (2007). Monte Carlo simulation to reconstruct formaldehyde exposure levels from summary parameters reported in the literature.. Ann Occup Hyg.

[r30] Lioy PJ, Freeman NC, Millette JR (2002). Dust: a metric for use in residential and building exposure assessment and source characterization.. Environ Health Perspect.

[r31] Lu C, Fenske RA, Simcox NJ, Kalman D (2000). Pesticide exposure of children in an agricultural community: evidence of household proximity to farmland and take home exposure pathways.. Environ Res.

[r32] McCauley LA, Lasarev MR, Higgins G, Rothlein J, Muniz J, Ebbert C (2001). Work characteristics and pesticide exposures among migrant agricultural families: a community-based research approach.. Environ Health Perspect.

[r33] McCauley LA, Michaels S, Rothlein J, Muniz J, Lasarev M, Ebbert C (2003). Pesticide exposure and self reported home hygiene: practices in agricultural families.. AAOHN J.

[r34] Morgan MK, Sheldon LS, Thomas KW, Egeghy PP, Croghan CW, Jones PA (2008). Adult and children’s exposure to 2,4-D from multiple sources and pathways.. J Expo Sci Environ Epidemiol.

[r35] Ritz B, Rull RP (2008). Assessment of environmental exposures from agricultural pesticides in childhood leukaemia studies: challenges and opportunities.. Radiat Prot Dosimetry.

[r36] Shields KF Bain RES Cronk R Wright JA Bartram J 2015 Association of supply type with fecal contamination of source water and household stored drinking water in developing countries: a bivariate meta-analysis. Environ Health Perspect 123 1222 1231, doi:10.1289/ehp.1409002 25956006 PMC4671240

[r37] Simcox NJ, Fenske RA, Wolz SA, Lee IC, Kalman DA (1995). Pesticides in household dust and soil: exposure pathways for children of agricultural families.. Environ Health Perspect.

[r38] Thompson B, Griffith WC, Barr DB, Coronado GD, Vigoren EM, Faustman EM (2014). Variability in the take-home pathway: farmworkers and non-farmworkers and their children.. J Expo Sci Environ Epidemiol.

[r39] Ward MH Lubin J Giglierano J Colt JS Wolter C Bekiroglu N 2006 Proximity to crops and residential exposure to agricultural herbicides in Iowa. Environ Health Perspect 114 893 897, doi:10.1289/ehp.8770 16759991 PMC1480526

[r40] Ward MH, Nuckols JR, Weigel SJ, Maxwell SK, Cantor KP, Miller RS (2000). Identifying populations potentially exposed to agricultural pesticides using remote sensing and a geographic information system.. Environ Health Perspect.

