# Supplementary material for: Relative Contributions of Agricultural Drift, Para-Occupational, and Residential Use Exposure Pathways to House Dust Pesticide Concentrations: Meta-Regression of Published Data
Source: Environ Health Perspect. 2016 Jul 26;125(3):296–305. doi: 10.1289/EHP426 (PMC5332194; doi:10.1289/EHP426)

Environ Health Perspect
DOI: 10.1289/EHP426

Note to readers with disabilities: *EHP* strives to ensure that all journal content is accessible to all readers. However, some figures and Supplemental Material published in *EHP* articles may not conform to 508 standards due to the complexity of the information being presented. If you need assistance accessing journal content, please contact ehp508@niehs.nih.gov. Our staff will work with you to assess and meet your accessibility needs within 3 working days.

Supplemental Material

Relative Contributions of Agricultural Drift, Para-Occupational, and Residential Use Exposure Pathways to House Dust Pesticide Concentrations: Meta-Regression of Published Data

Nicole C. Deziel, Laura E. Beane Freeman, Barry I. Graubard, Rena R. Jones, Jane A. Hoppin, Kent Thomas, Cynthia J. Hines, Aaron Blair, Dale P. Sandler, Honglei Chen, Jay H. Lubin, Gabriella Andreotti, Michael C. R. Alavanja, and Melissa C. Friesen

Table of Contents

Figure S1 GM house dust pesticide concentrations decreased logarithmically with distance between home and treated fields, by pesticide type.
Figure S2 GM house dust pesticide concentrations decreased logarithmically with distance between home and treated fields for each unique paper/pesticide active ingredient.

Additional Files

Supplemental Code and Data ZIP File

Appendix 1--SAS Code and Data

Figure S1 GM house dust pesticide concentrations decreased logarithmically with distance between home and treated fields, by pesticide type. Solid line = predicted association from meta-regression models. Circles = Distance/Pesticide/Paper-specific GMs, with circle width = $(\log GSD)^2/N$.

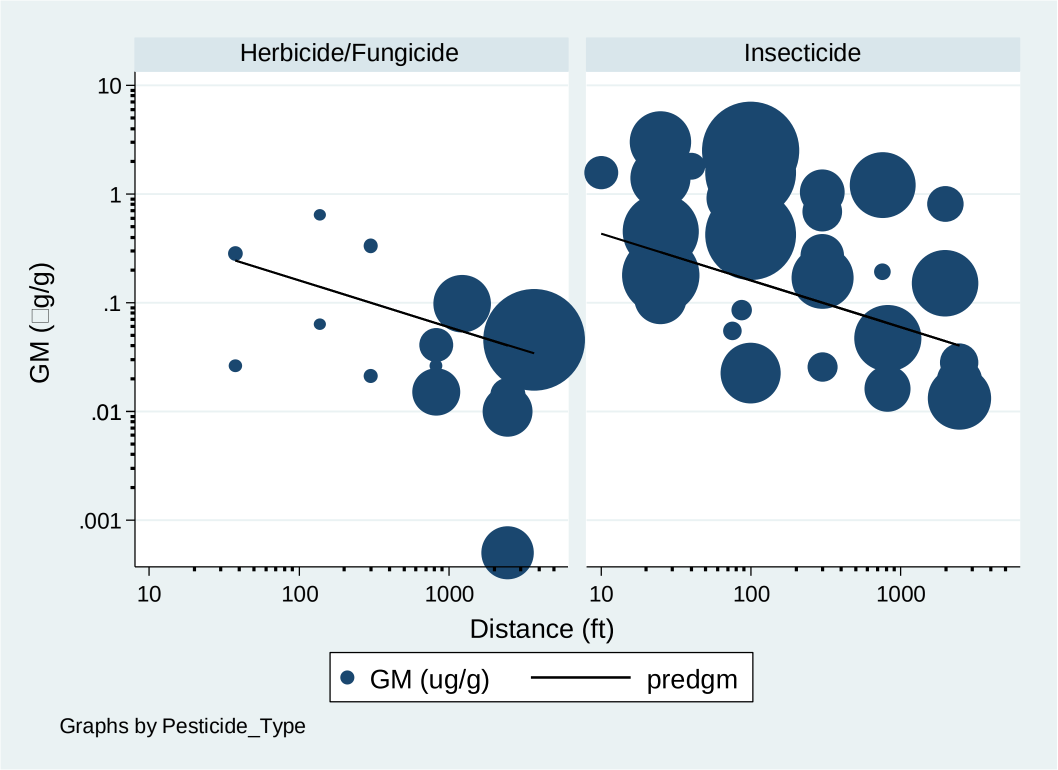

Figure S2 **GM house dust pesticide concentrations decreased logarithmically with distance between home and treated fields for each unique paper/pesticide active ingredient. Black line = predicted association from overall model. Solid Line = predicted association for the given data points. Circles = Distance/Pesticide/Paper-specific ln(GM)s, with circle width = $(\log GSD)^2/N$.**

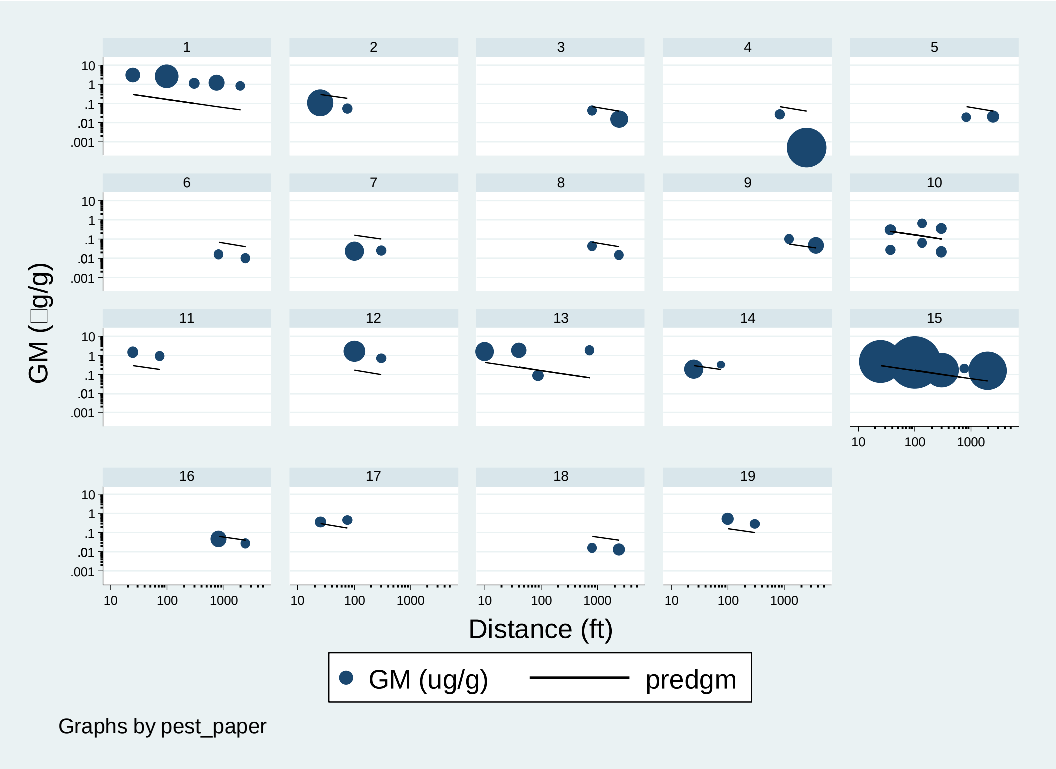

Supplement: (95 KB) PDF [file EHP426.s001.acco.pdf]
